# Optimization of the cyclotide framework to improve cell penetration properties

**DOI:** 10.3389/fphar.2015.00017

**Published:** 2015-02-09

**Authors:** Yen-Hua Huang, Stephanie Chaousis, Olivier Cheneval, David J. Craik, Sónia T. Henriques

**Affiliations:** Institute for Molecular Bioscience, The University of QueenslandBrisbane, QLD, Australia

**Keywords:** cyclic cell-penetrating peptide, cyclotide, peptide scaffold, drug delivery, peptide reengineering

## Abstract

Cell penetrating peptides have been regarded as promising vectors to deliver hydrophilic molecules inside cells. Although they are great tools for research and have high potential as drug delivery systems, their application as drugs is impaired by their low stability in serum. Cyclotides, cyclic disulfide-rich peptides from plants, are ultra-stable molecules that have inspired applications in drug design as they can be used as scaffolds to stabilize linear bioactive sequences. Recently, they have also been shown to possess cell-penetrating properties. The combination of their remarkable stability and cell-penetrating properties opens new avenues for the application of peptides to bind to and inhibit intracellular proteins. Nevertheless, for a broader application of these molecules as vectors is of utmost importance to improve their cellular internalization efficiency. In this study we successfully modified MCoTI-II, one of the most widely studied cyclotide scaffolds in drug design, and improved its internalization properties. The internalization of the newly designed MCoTI-II is as efficient as the gold standard cell-penetrating peptide (CPP) TAT and maintains all the required features as a template to graft desired bioactivities.

## Introduction

Despite their high selectivity, potency and low toxicity, peptides have in the past been discounted as good therapeutics due to their poor stability and low delivery efficiency. However, recent advances that improve their stability have stimulated interest in pursuing peptides as alternative therapeutics. In particular, the discovery of stable disulfide-rich peptides with high potency, specificity and tolerance to sequence modification have provided strong support for the use of peptides as therapeutic templates (Craik et al., 2013). Examples include disulfide-rich toxins isolated from the venoms of cone snails, which have exquisite selectivity for membrane receptors (Schroeder and Craik, 2012).

The peptide field also received renewed interest upon the discovery of positively-charged sequences, named cell-penetrating peptides (CPPs), able to carry and translocate large proteins or other hydrophilic molecules, into cells (Henriques et al., 2006). Although a proven research tool, CPPs still have limitations for therapeutic protein delivery due to the low enzymatic stability of the CPP protein conjugates, a drawback in both oral and injection delivery.

**Figure 1:**
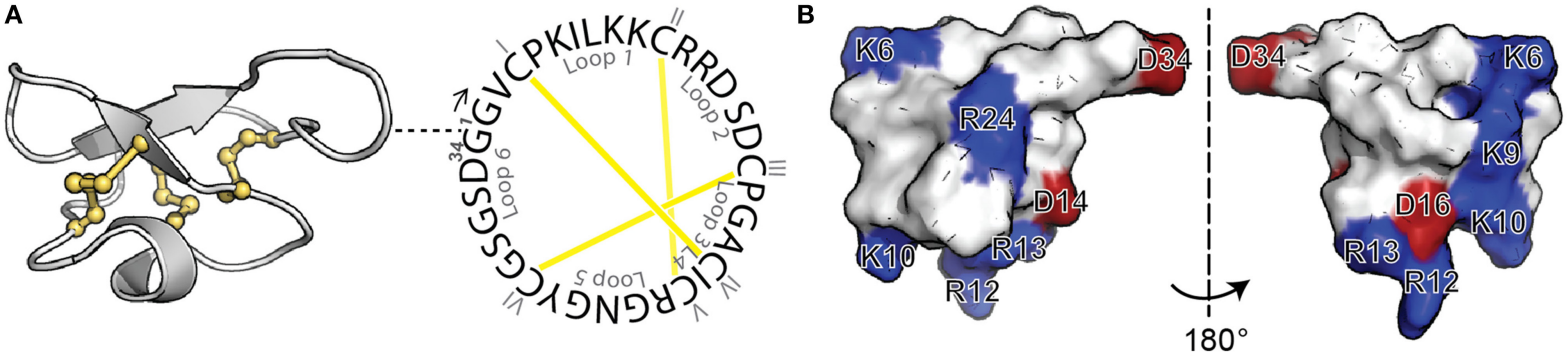
**Structure of MCoTI-II**. **(A)** Three-dimensional structure (left; PDB ID: lib9) and sequence of native MCoTI-II (right) showing the cyclic backbone and the three disulfide bonds (shown in yellow) arranged in a knot, the signature features of cyclotides. The position of the first residue (G1) is shown in the three-dimensional structure with a dashed line. The backbone segments between Cys are named loops 1–6 and the Cys residues are numbered with I–VI. **(B)** Surface representation of MCoTI-II shown in two views. Positively-charged (blue) and negatively-charged (red) residues are labeled.

Cyclotides are a fascinating class of cyclic disulfide-rich peptides that have inspired applications of peptides in drug design. Isolated from plants, cyclotides are characterized by a cyclic peptide backbone together with three-disulfide bonds arranged in a knot, referred to as a cyclic cystine knot (CCK) motif (Figure 1A) (Craik et al., 1999). This structural motif confers cyclotides with remarkable stability, which combined with their amenability to modification, has stimulated their use as a drug scaffold (Henriques and Craik, 2010; Poth et al., 2013). Specifically, the backbone portions between cysteine residues, referred to as loops, can be modified to incorporate foreign bioactive peptide sequences. The grafting of these bioactive regions confers cyclotides with specific activities without disturbing the overall fold and can be used to inhibit therapeutic targets.

Cyclotides have been successfully modified to incorporate a wide range of desired activities (Poth et al., 2013). Examples include reengineered cyclotides with angiogenic activity and potential wound healing properties (Chan et al., 2011), peptides that target melanocortin receptors with the potential to treat obesity (Eliasen et al., 2012), molecules with immuno-regulatory activity and molecules with capacity to target multiple sclerosis (Wang et al., 2014), as well as peptide-based inhibitors of matriptase, a target for breast cancer (Quimbar et al., 2013). All these modified cyclotides were designed to target extracellular or membrane-bound receptors.

Interestingly, some cyclotides have been found to possess cell-penetrating properties (Greenwood et al., 2007; Cascales et al., 2011; Contreras et al., 2011). The identification of such a property suggests that cyclotides can be used, not only as a template to stabilize bioactive peptide sequences, but also as a vector to introduce the bioactive sequences inside cells. This potential suggests that reengineered cyclotides could be used to inhibit intracellular protein/protein interactions and further extend their potential application as therapeutics. This promising potential has been recently realized with a pioneering work by Camarero's group, in which an 18-amino acid sequence was grafted into loop 6 of *Momordica cochinchinensis* trypsin inhibitor I (MCoTI-I) and shown to inhibit the intracellular interaction of p53 with the oncoprotein Hdm2 *in vitro* and in a mouse model (Ji et al., 2013).

So far, MCoTI-I and closely related MCoTI-II are the cyclotides that have been extensively studied for their internalization properties, and also two of the preferred scaffolds in drug design (Poth et al., 2013). Nevertheless, their internalization efficiency, when compared with typical linear CPPs, is rather low (D'Souza et al., 2014). Our hypothesis is that, for an efficient and broad application of cyclotides as frameworks to deliver bioactive sequences into cells, it is of major importance to improve the internalization efficiency of MCoTI-II. Therefore, in the current study we have modified MCoTI-II to improve the intracellular uptake, without compromising the low toxicity and ability to be a good template for drug design.

## Materials and methods

### Peptide synthesis and labeling

MCoTI-II and its analogs were synthesized using manual solid phase peptide synthesis (SPPS) based on Boc-chemistry as described previously (Chan et al., 2011), but using 2-(6-chloro-1H-benzotriazole-1-yl)-1,1,3,3-tetramethylaminium hexafluorophosphate (HCTU) as the coupling reagent. Peptides were cleaved from the resin using hydrogen fluoride with the scavenger *p*-cresol at 0°C for 1 h. Crude peptides were purified using Phenomenex C18 columns on reversed-phase high-performance liquid chromatography (RP-HPLC). Cyclization and oxidation of the peptides was achieved by incubating the linear reduced peptides in 0.1 M ammonium bicarbonate (pH 8.5) for 24 h and the resulting peptides were purified using RP-HPLC with 1% gradient of 0–80% of solvent B (acetonitrile 90% (v/v) with 0.045% (v/v) trifluoroacetic acid (TFA) in H_2_O) in solvent A (0.05% (v/v) TFA in H_2_O) to >95% purity. The mass of peptides was confirmed using electrospray ionization mass spectrometry (ESI-MS) and the purity of peptides was verified using an ultra high performance liquid chromatography system (UHPLC, Shimadzu) with 4%/min gradient of 5–35% solvent B on an analytical C18 column (RRHD 2.1 × 50 mm, 1.8 μm, Agilent). Linear peptides TAT (NH_2_-YGRKKRRQRRRPPQG-COOH) and modified CTP512 with one of the Arg residues replaced with a Lys residue for labeling purpose (NH_2_-YGRKARRRRRR-COOH) were synthesized using SPPS based on Fmoc-chemistry on an automated peptide synthesizer (Symphony®, Protein Technologies) and cleaved as described previously (Cheneval et al., 2014).

Peptides were labeled with Alexa fluor® 488 sulfodichlorophenol ester (Life Technologies) through the side-chain amine of Lys residue as described before (Torcato et al., 2013a). The retention time and the purity of labeled peptides were determined using UHPLC. The mass of peptides was confirmed using ESI-MS. Quantification of singly-labeled peptides was performed using Lambert-Beer law with the absorbance of the dye at 495 nm (ε_495_ = 71,000 M^−1^cm^−1^) (D'Souza et al., 2014). Non-labeled peptides were quantified following absorbance at 280 nm (ε_280_ = 1865 M^−1^cm^−1^ for MCoTI-II and MCo-RM1, and 3355 M^−1^cm^−1^ for MCo-CTP).

### Cell culture

Adherent human cervix epitheloid carcinoma (HeLa) cells were grown in Dulbecco's modified Eagle's medium (DMEM; supplemented with 1% (v/v) penicillin/streptomycin and 10% (v/v) of fetal bovine serum) at 37°C in humidified atmosphere containing 5% CO_2_. Cells were split every 2 days or after reaching confluence with ¼ dilution. HeLa cells were seeded in a 24-well plate with 1 × 10^5^ cells per well the day before the internalization assay.

### Internalization assay

Internalization of fluorescently-labeled peptides into HeLa cells was followed by flow cytometry as described previously (Torcato et al., 2013a; D'Souza et al., 2014). Briefly, Alexa fluor® 488 labeled TAT, CTP512, MCoTI-II, MCo-RM1 and MCo-CTP at concentrations varying from 0.25 to 8 μM were incubated with HeLa cells for 1 h at 37°C in medium without serum. Non-internalized peptides were washed with cold phosphate buffer solution (PBS) and cells were harvested from the plate by trypsinization, spun at 1500 rpm at 4°C and resuspended in cold PBS. Cell mean fluorescence emission was measured by flow cytometry (BD FACSCanto™ II) by analyzing 10,000 cells per sample with excitation at 488 nm and emission at 530/30 nm, before and after the addition of trypan blue (TB; 160 μg/mL). Experiments were repeated on three independent days.

### Cytotoxicity assay

Toxicity to HeLa cells induced by non-labeled peptides was examined with 2-fold dilutions starting from 64 μM and quantified using a resazurin assay as detailed before (Torcato et al., 2013b). Experiments were done in duplicate.

### Serum stability

The stability of peptides in human serum was examined using a protocol reported previously (Chan et al., 2011). Briefly, stock solutions of peptides (300 μM) were diluted 10 times with pre-warmed 100% human serum isolated from male AB plasma (Sigma-Aldrich) and incubated at 37°C for 0, 1, 2, 3, 5, 8, 12 and 24 h. Controls with peptides in PBS were included. The reaction was stopped by denaturing the serum proteins with urea at a final concentration of 3 M at 4°C for 10 min, followed by precipitation of serum proteins with trichloroacetic acid at a final concentration of 7% (v/v) (4°C, 10 min) and centrifugation (17,000 g, 10 min). The supernatant of each sample was recovered and run on an analytical column using a linear gradient of 5–30% solvent B (acetonitrile 90% (v/v) with 0.045% (v/v) TFA in H_2_O) in solvent A (0.05% (v/v) TFA in H_2_O) over 25 min at a flow rate of 0.3 mL/min with monitoring at 215 nm. The elution profile of each peptide was identified by the PBS sample from 0 time point. The percentage of peptide remaining in serum-treated samples was determined by comparing the height of the peptide peak obtained at each time point with that of the peptide peak obtained at 0 time point. Each experiment was done in triplicate.

### ^1^H NMR spectroscopic characterization

Lyophilized peptides (purity > 95%) were dissolved in 10% (v/v) D_2_O to a final concentration of 1 mM. One- and two-dimensional TOCSY (total correlation spectroscopy) and NOESY (nuclear overhauser effect spectroscopy) spectra of MCoTI-II, MCo-RM1 and MCo-CTP were acquired using an Avance-600 MHz spectrometer (Bruker) at 25°C. The mixing time was 80 ms and 200–300 ms for TOCSY and NOESY, respectively. Spectra were internally referenced to 2,2-dimethyl-2-silapentane-5-sulfonic acid (DSS) at 0.00 ppm and analyzed using SPARKY (version 3.114). The backbone secondary chemical shifts of peptides were calculated for each residue using the random coil chemical shifts (Wishart et al., 1995).

## Results

### Reengineering the cyclotide framework

Previous studies on MCoTI-II and its analogs have shown that the overall positive charge and the distribution of the basic residues on the surface of the molecule (Figure 1B) are important for its cellular internalization (Cascales et al., 2011; D'Souza et al., 2014). In general it has been shown that Arg residues, compared to Lys residues, improve translocation of CPPs (Fuchs and Raines, 2006; Sundlass and Raines, 2011). The guanidinium group of Arg is able to form additional hydrogen bonds, compared to the ammonium group of Lys, and therefore more readily binds to carboxyl, phosphoryl and sulfuryl groups at the cell surface. Thus, we first designed an analog, MCo-RM1 (Table 1), in which all the Lys residues in the positive patch (see Figure 1B) were replaced with Arg residues. In this analog, Arg 24 in loop 5 was also replaced with a Lys. The purpose of this modification was to introduce a Lys side chain for the fluorescent labeling required for the internalization studies and to have the fluorescent probe away from the positively-charged patch.

**Table 1:** **Sequences of MCoTI-II and its analogs used in this study**.

**Peptide**	**Loop 6**	**I**	**Loop 1**	**II**	**Loop 2**	**III**	**Loop 3**	**IV**	**4**	**V**	**Loop 5**	**VI**	**Loop 6**	**Charge^a^**
MCoTI-II	GGV	**C**	PKILKK--	**C**	RRDSD----	**C**	PGA	**C**	I	**C**	RGNGY	**C**	GSGSD	+3
MCo-RM1	GGV	**C**	P**R**IL**RR**--	**C**	RRDSD----	**C**	PGA	**C**	I	**C**	**K**GNGY	**C**	GSGSD	+3
MCo-RM2	GGV	**C**	P**R**IL**RR**--	**C**	RR**R**S**R----**	**C**	PGA	**C**	I	**C**	RGNGY	**C**	GSGS**K**	+9
MCo-RM3	GGV	**C**	PRILRR--	**C**	RR**ARRRARR**	**C**	PGA	**C**	I	**C**	RGNGY	**C**	GSGS**K**	+12
MCo-CTP	GGV	**C**	**YGRRARRR**	**C**	RRDSD----	**C**	PGA	**C**	I	**C**	**K**GNGY	**C**	GSGSD	+5

a *Overall peptide charge at pH 7.4*.

MCo-RM2 and MCo-RM3 were designed to increase the overall charge. All the acidic residues in MCo-RM2 were replaced with basic residues (Asp to Arg in loop 2 and Asp to Lys in loop 6), whereas the loop 2 of MCo-RM3 was replaced with a longer sequence containing extra Arg residues and two Ala residues intermediating the Arg residues to potentially help in the peptide assembly and folding. Both MCo-RM2 and MCo-RM3 have the Asp residue in loop 6 replaced with a Lys. This modification was included to further increase the overall charge of these peptides and to place the fluorescent label away from the positively-charged patch.

MCo-CTP includes a portion of a previously identified CPP sequence, CTP512 (Kim et al., 2006). This sequence, based on TAT, was shown to be better internalized and to have higher distribution within the cytoplasm than TAT. Because TAT is a nuclear localization sequence, it is likely to direct fused proteins toward the nucleus; therefore, CTP512 was designed to retain the ability to translocate through the cell membrane but to remain in the cytoplasm after internalization. MCo-CTP incorporates a sequence derived from CTP512 into loop 1 of the MCoTI-II scaffold and a Lys for labeling into loop 5 (Lys 26, see Table 1).

### Synthesis, overall fold and tridimensional structure of reengineered peptides

Novel designed MCoTI-II peptides were assembled using SPPS based on Boc-chemistry and cyclized and oxidized with ammonium bicarbonate buffer at pH 8.5. Purification with acetonitrile gradient and a first assessment of folding were conducted using RP-HPLC and ESI-MS; chromatograms of both MCo-RM1 and MCo-CTP showed typical profiles of cyclized and oxidized cyclotides, whereas the chromatograms obtained for MCo-RM2 and MCo-RM3 showed multiple peaks corresponding to misfolded isomers. Folding trials with various conditions were conducted, but no improvement in the folding was achieved. Thus, additional studies with MCo-RM2 and MCo-RM3 could not be performed. Details on the characterization of the peptides (i.e., UHPLC retention time, purity percentage and the observed m/z values) are included in Supplementary Table 1.

**Figure 2:**
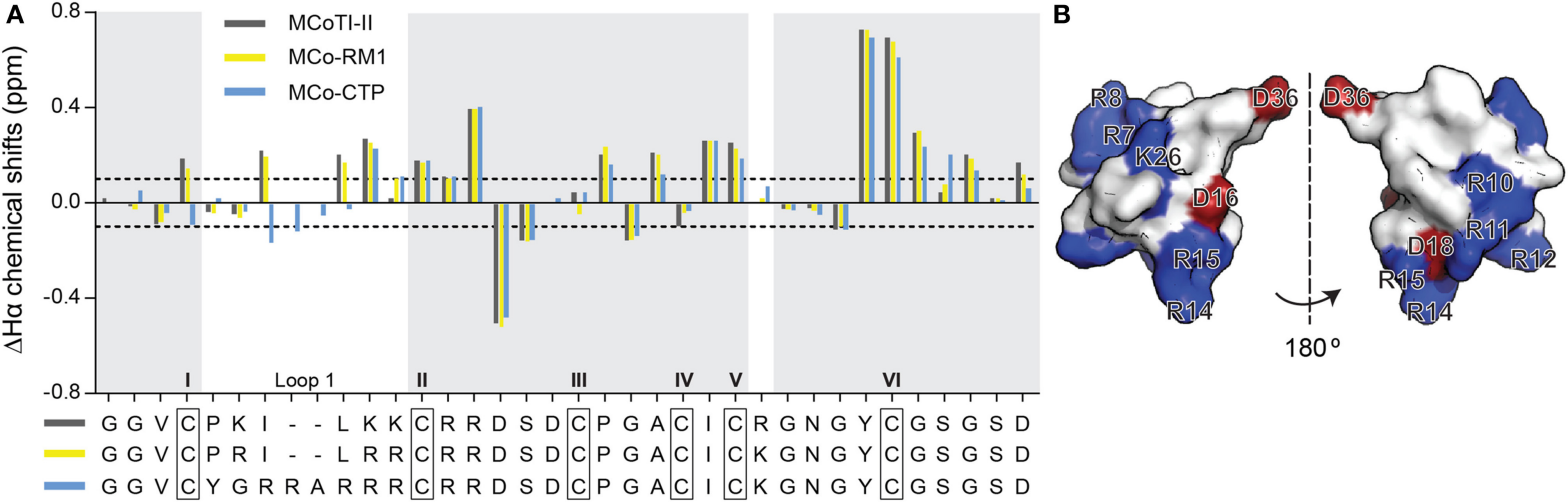
**Structure of MCoTI-II analogs. (A)** Secondary α-proton chemical shifts of MCoTI-II, MCo-RM1, and MCo-CTP determined by two-dimensional NMR spectroscopy. The observed secondary chemical shifts of MCoTI-II, MCo-RM1 and MCo-CTP are plotted as dark gray, yellow and blue bars, respectively. The peptide sequences are shown below the graph. Regions with identical sequences are shaded in light gray. The six Cys residues are highlighted with black boxes and labeled with Roman numerals I–VI. The dotted lines indicate the shift changes at 0.1 and –0.1 ppm. **(B)** Surface structure representation of MCo-CTP, shown in two views (flipped by 180°), as modulated based on MCoTI-II structure (PDB ID: lib9) using modeler and PyMOL. Positively-charged and negatively-charged residues are shown in blue and red, respectively.

After purification of MCo-RM1 and MCo-CTP, the overall fold of the peptides was evaluated by ^1^H NMR spectroscopy. The secondary α-proton chemical shifts of MCo-RM1 and MCo-CTP in the gray-shaded regions of Figure 2A are comparable to those of native MCoTI-II. Differences in chemical shifts are only evident in loop 1, where residues were substituted, and the flanking regions. These results suggest that the overall structure of the designed peptides is similar to that of the native scaffold. The three-dimensional (3D) structure of the engineered MCo-CTP was predicted using Modeler 9v2 (Sali and Blundell, 1993) based on the known 3D structure of MCoTI-II (PDB: 1IB9) (Felizmenio-Quimio et al., 2001), and its surface structure was generated using PyMOL Molecular Graphics System (Version 1.5.0.4 Schrödinger, LLC; www.pymol.org). Figure 2B shows that the newly designed molecule MCo-CTP contains a positively-charged patch at the surface.

### Internalization into HeLa cells

**Figure 3:**
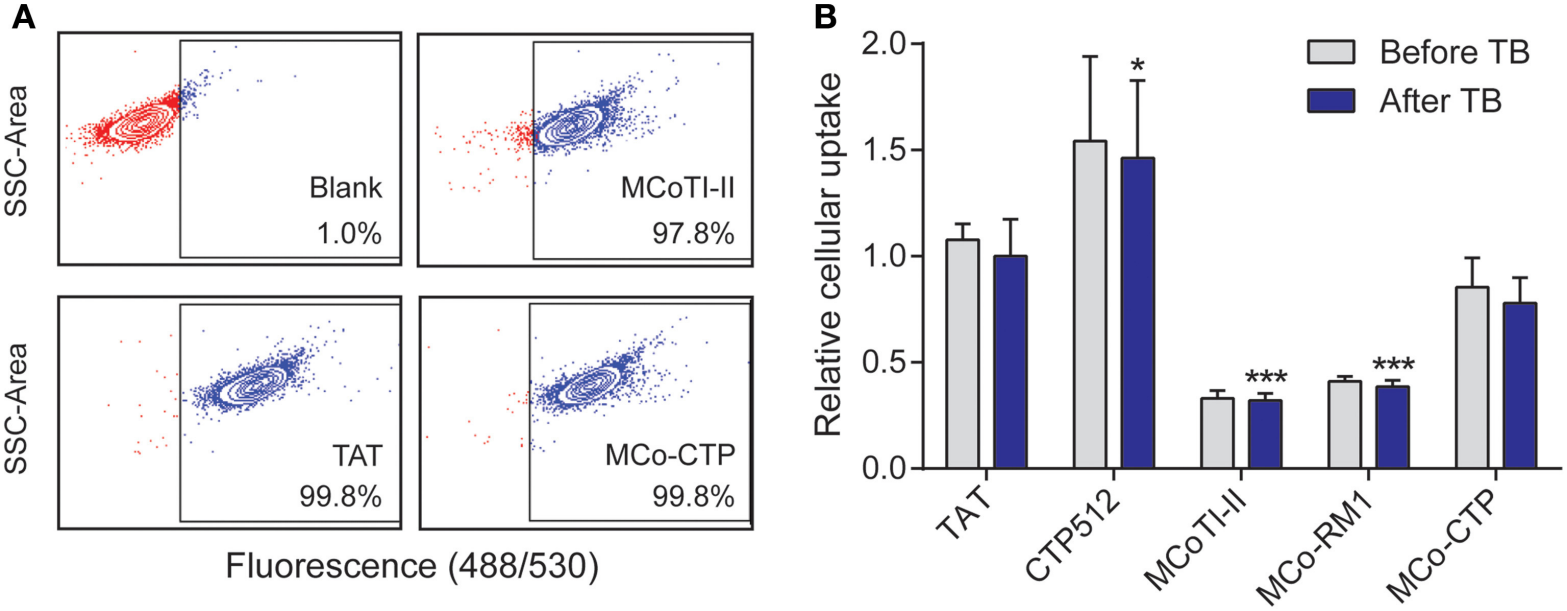
**Internalization of peptides into HeLa cells**. Cells were treated with 8 μM of fluorescently-labeled peptides for 1 h at 37°C and the internalization was measured by fluorescence emission of Alexa fluor® 488 using flow cytometry [excitation at 488 nm and detection at 530 nm (band pass 30 nm)]. The fluorescence emission was measured before and after the addition of trypan blue (TB; 160 μg/mL); 10,000 cells were analyzed per sample. **(A)** Dotplots showing the side scatter (SSC) as function of the fluorescence emission intensity obtained after addition of TB; both axes are in log scale. The gate of fluorescent cells was drawn with the blank (without peptide) to exclude dark events. **(B)** Mean fluorescence intensity obtained with peptide-treated cells before and after addition of TB. The fluorescence signal was normalized to TAT after addition of TB. Data are represented as mean ± SD of three independent experiments (see values in Table 2); the statistical analysis was performed by One-Way ANOVA by comparison with TAT (^*^p < 0.05, ^***^p < 0.001).

The internalization efficiency of the various peptides was measured by comparing their uptake into HeLa cells. All the peptides were conjugated with a single Alexa fluor® 488 molecule and had a purity of >93%, as confirmed by ESI-MS and UHPLC (see Supplementary Table 1). HeLa cells were treated with a range of concentrations, varying from 0.25 to 8 μM, of each labeled peptide for 1 h at 37°C and the percentage of fluorescent cells and mean fluorescence emission intensity of Alexa fluor® 488 measured by flow cytometry as before (Torcato et al., 2013a) (Figure 3).

Virtually all the cells became fluorescent upon treatment with 8 μM of the tested peptides (the percentage of fluorescent cells was >97%, Figure 3A), confirming the ability of MCoTI-II and its analogs to target HeLa cells. The percentage of fluorescent cells did not change after the addition of trypan blue (TB), an aqueous fluorescence quencher unable to enter viable cells. This observation confirms that the internalization occurred through a non-permeabilizing mechanism (Torcato et al., 2013a). Furthermore, the mean fluorescence emission did not have a significant drop after the addition of TB (Figure 3B and Table 2). The fact that the fluorescent peptides were inaccessible to the aqueous environment suggests that the peptides were not cell surface-associated but internalized inside the cell. This is in agreement with previous studies on MCoTI-II that showed it has only a weak ability to bind to model lipid membranes (Cascales et al., 2011; D'Souza et al., 2014).

**Table 2:** **Fluorescence signal of HeLa cells after treatment with labeled peptides^a^**.

**Peptide**	**Before trypan blue**	**After trypan blue**
TAT	1.08 ± 0.07	1.00 ± 0.17
CTP512	1.54 ± 0.40	1.46 ± 0.36
MCoTI-II	0.33 ± 0.04	0.32 ± 0.03
MCo-RM1	0.41 ± 0.02	0.39 ± 0.03
MCo-CTP	0.85 ± 0.14	0.78 ± 0.12

a *Cells were treated with 8 μM of Alexa fluor® 488 -labeled peptides for 1 h at 37° C and the mean fluorescence emission intensity of 10,000 cells was measured using flow cytometry. The mean fluorescence emission signal was normalized for the signal obtained with TAT after treatment with trypan blue*.

The uptake efficiency of the tested peptides was ranked by comparison of the mean fluorescence emission obtained after the addition of TB, and followed the trend: MCoTI-II < MCo-RM1 < MCo-CTP ~ TAT < CTP512. MCo-RM1 has a 22% improvement in internalization efficiency compared to its parent peptide MCoTI-II, whereas MCo-CTP has a 2.4-fold increase and has equivalent internalization efficiency to TAT (see Table 2 and Figure 3B). Although the observed internalization of MCo-CTP was lower than that of CTP512, the grafting of a linear CPP sequence into MCoTI-II scaffold improves its internalization efficiency.

### Toxicity and stability of novel peptides

**Figure 4:**
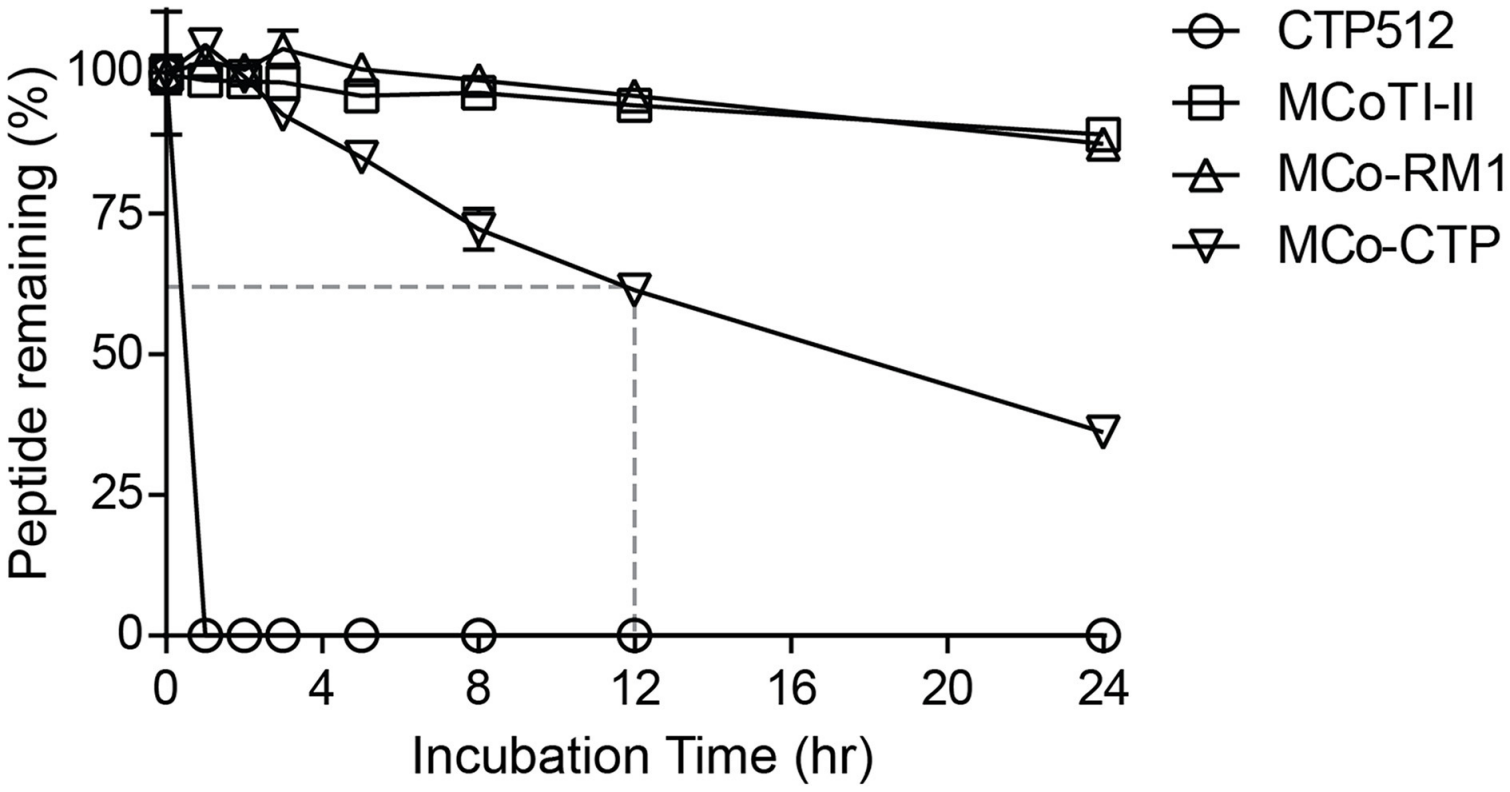
**Serum-stability of tested peptides**. Percentage of peptide remaining upon incubation with human serum. The portion of serum-treated peptide remaining was determined by calculating the height of the respective elution peak in analytical-HPLC and comparing it with control without incubation. Data points are means ± SD of three experiments.

None of the peptides showed cytotoxicity against HeLa cells at concentrations up to 64 μM, as assayed using a resazurin test (data not shown). To evaluate whether the designed peptides retained the stability of the native MCoTI-II scaffold, they were incubated with human serum and the portion of peptide remaining was quantified by analytical-HPLC. Figure 4 shows that the linear CTP512 was fully degraded by proteases in serum within 1 h, whereas MCoTI-II and all the analogs were stable for several hours. Unlike MCo-RM1, MCo-CTP is less stable than its parent peptide MCoTI-II; nevertheless, more than 60% of the peptide remained intact after 12 h incubation in serum. These results confirm that the CCK structure prevents enzymatic degradation of grafted sequences, even those containing a large number of basic residues.

## Discussion

Arising from the success of MCoTI-II as a novel scaffold for drug design (Poth et al., 2013), in the present study we were interested in improving the uptake of this peptide for its more efficient use as a drug template and delivery system to inhibit intracellular targets. Loop 6 of this peptide is normally preferred to graft desired bioactive sequences as it is the longest and most flexible loop in MCoTI-II and therefore the most likely to keep the conformation of foreign sequences. With this in mind we tried to improve the cell-penetrating properties of MCoTI-II without modification of loop 6.

The rationale behind the designed peptides was to improve the positively-charged patch at the surface of MCoTI-II, and this was proved to be a successful strategy, as the uptake efficiency increased by ~20% for MCo-RM1 and to ~240% for MCo-CTP. The analog MCo-RM2, in which all the negatively-charged residues were replaced with basic residues, did not fold, suggesting that the negatively-charged residues in loop 2 are important to keep the overall structure of the scaffold and probably help form the correct disulfide connectivity. This shows that, although positive charges are required for the internalization of MCoTI-II, a balance between positive and negatively-charged residues is required to efficiently fold the MCoTI-II structure.

Both MCo-RM1 and MCo-CTP kept the overall structure of MCoTI-II and were stable in human serum. Grafting the CTP512 into the MCoTI-II scaffold strikingly increases the stability of this linear CPP, as shown with MCo-CTP. This clearly shows that using a cyclotide scaffold as a strategy to translocate bioactive molecules into cells is a desirable alternative to linear CPPs. It is worth mentioning that for the purpose of labeling, the MCo-CTP has its Arg in loop 5 replaced with a Lys, but for potential therapeutic applications, as labeling will not be required, the original Arg could be kept, as this might improve its uptake efficiency. Without the label the overall charge of MCo-CTP is higher, so the overall uptake is expected to be higher than that of the labeled peptide shown in this study.

Previous mechanistic studies of MCoTI-I and MCoTI-II suggested that these peptides enter cells via endocytic pathways and lack membrane-binding properties (Cascales et al., 2011; Contreras et al., 2011; D'Souza et al., 2014). Although mechanistic studies were not within the scope of the current study, based on the internalization results we postulate that the designed peptides do not bind strongly to membranes and probably enter cells following the same mechanism as the native MCoTI-II.

**Figure 5:**
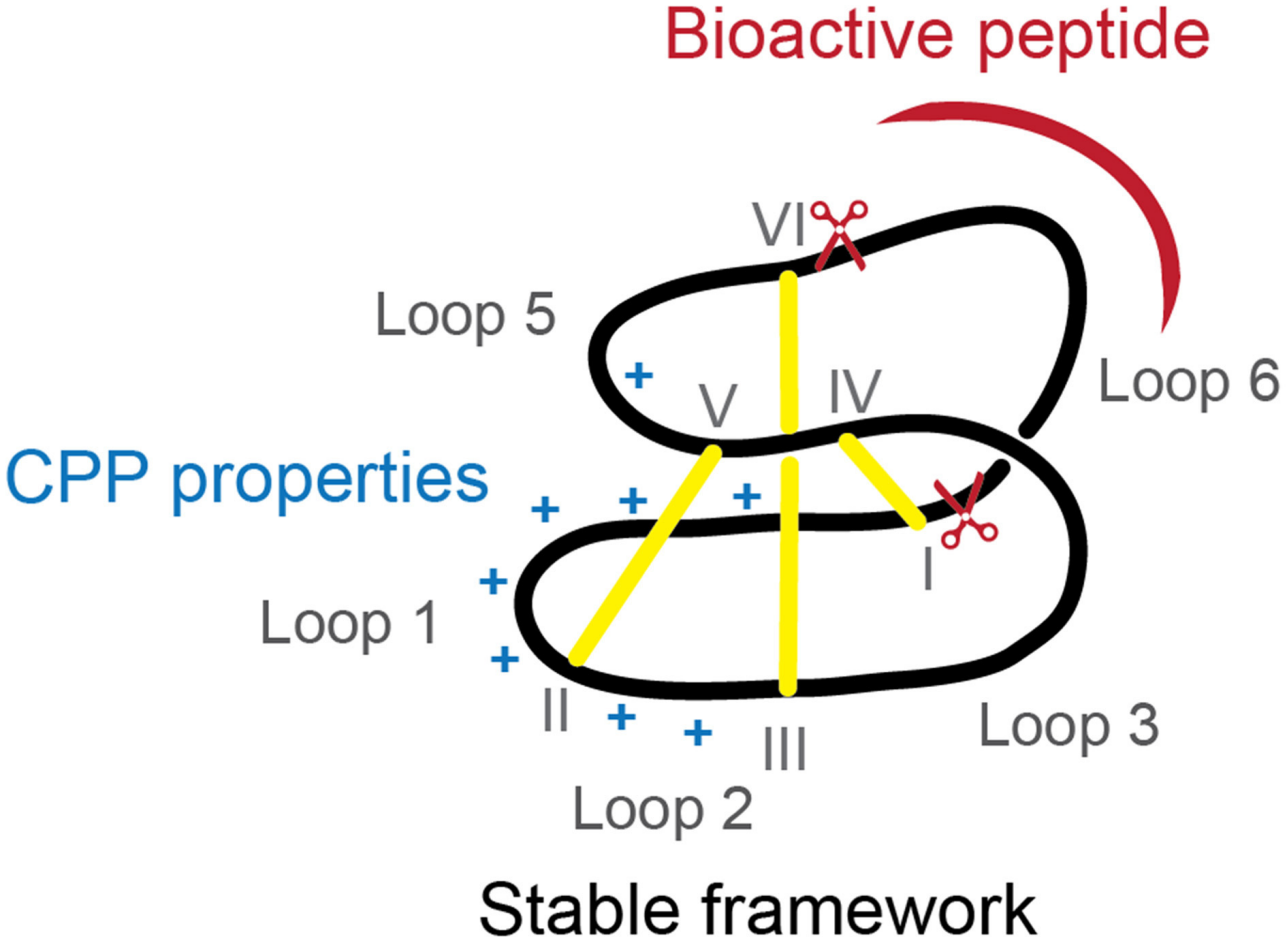
**Diagram of ameliorated cyclotide scaffold**. The modified cyclotide retains the overall fold, stability and loop 6 to graft bioactive linear sequences (represented in red) but has improved cellular uptake due to incorporation of a cell-penetrating peptide with extra positive charges (represented in blue).

Intracellular protein-protein interactions are traditionally considered “undruggable” as protein-protein interfaces are too large to be inhibited by small-molecule approaches and not accessible to antibodies, unable to cross the cell membrane. Nevertheless, new developments in the peptide field have shown that inhibition of intracellular proteins is possible and hidden drug targets are now starting to be explored (Hoe et al., 2014). In the current study we have shown that cyclotides can be modified to improve their internalization, while maintaining loop 6 (preferred for grafting) intact and high resistance to protease degradation. The reengineered MCoTI-II (Figure 5) with improved cellular uptake can be used as a novel cyclotide scaffold to stabilize a bioactive linear peptide and deliver it efficiently inside the cells to inhibit intracellular protein-protein interactions.

### Conflict of interest statement

The authors declare that the research was conducted in the absence of any commercial or financial relationships that could be construed as a potential conflict of interest.
