# Supplementary material for: Optimization of the cyclotide framework to improve cell penetration properties
Source: Front Pharmacol. 2015 Feb 9;6:17. doi: 10.3389/fphar.2015.00017 (PMC4321561; doi:10.3389/fphar.2015.00017)
Supplement: Supplementary file 1 [file Table1.PDF]

Supplementary Table 1. The retention time, average mass, observed m/z values from ESI-MS and the percentage of purity of peptides synthesized for this study.

Peptide	Retention time (min)	Average mass (Da)	Observed m/z from ESI-MS			Purity (%)
			2+	3+	4+	
MCoTI-II	3.545	3453.0	1727.1	1151.7	864.1	99.1
MCo-RM1	2.776	3509.0	1755.2	1170.4	878.2	97.5
MCo-CTP	1.847	3789.3	-	1264.3	948.2	99.4
CTP512	0.538	1530.8	766.3	511.4	-	98.5
TAT	0.907	1939.3	969.9	647.1	485.7	99.3
MCoTI-II*	3.666	3968.5	1985.1	1323.8	993.1	95.9
MCo-RM1*	2.864	4024.5	-	1342.5	1007.1	96.0
MCo-CTP*	2.484	4304.8	-	1435.9	1077.2	93.9
CTP512*	1.188	2046.3	1024.3	683.4	512.8	99.0
TAT*	1.384	2454.8	1228.7	819.5	614.9	99.2

*Peptide labeled with one Alexa fluor® 488 molecule
